# Annotations

**Published:** 1905-12-30

**Authors:** 


### ANNOTATIONS.

### The Tyranny of the Telephone.

The question whether the use of the telephone is an unmixed advantage is asked this week by a correspondent of the *Times*, who, having affirmed that "it requires a great deal of answering," proceeds to say that the stockbroker, the lawyer, the house agent, the keen man of business, would have one answer to give, "while the doctors might possibly have another." Our contemporary, commenting on this communication, deals with the subject from the business point of view, and, though admitting the worries which are caused by the telephone, affirms, in effect, that the people who complain of them would be the first to deplore the disappearance of the instrument. This is probably quite true, but with regard to the opinion of doctors, it is not altogether favourable. They have felt the tyranny of the telephone, groaned under it, suffered from it, and would be glad to obtain relief from it. In any circumstances there is little peace for the medical man, for sick persons and their relatives are often lacking in consideration; but the constant resort to the telephone on the part of patients whose illness is often more imaginary than real, has greatly added to the doctor's worries without augmenting his income. How to remedy the evil is another matter; and we are afraid that unless or until a code of ethics of the telephone is recognised and as scrupulously observed as any other amenities of modern society, it is likely to increase rather than to diminish.

### Vagrancy as a Disease.

Doubtless one of the many social problems which will tax the energy and wisdom of the recently appointed Poor-law Commission is that of vagrancy. Now, as always, the vagrant is ubiquitous. As soon as the halcyon days of the monasteries were over, a persistent persecution of the beggar began; but in spite of the harshest and most cruel laws he still continued to prowl over the country and levy his annual tax upon the benevolent, until at last, seemingly from sheer weariness of effort, the public have come to regard the vagrant as an evil which must be patiently endured. It is true that vagrancy is officially regarded as beyond the pale of the law-abiding citizen; nevertheless the fault is esteemed lightly, and, indeed, is perhaps rather fostered than retarded by certain details in the administration of relief under the Poor Law. The evils of vagrancy are sufficiently well known. The wandering beggar is a frequent carrier of contagion and a potent factor in the distribution of small-pox and other infectious disorders. Hence he ought to be regarded not merely as a useless burden, but as an actual menace to society. Lunatic asylums were originally established not on behalf of the sufferers from mental disease, but rather to protect the public from the irresponsible actions of the insane. None the less these institutions have permitted scientific study of the mentally afflicted, and have resulted in discoveries which have proved to be of the utmost benefit to both the patients and the public. Is it too much to hope that the collective treatment of vagrancy may soon be adopted with similar beneficial results? Medical officers of workhouses and prisons have stated that vagrancy is a disease and should be treated as such, and that to enforce penalties for it as though it were a crime is as unreasonable as to treat the lunatic by similar methods. This, we think, is the keynote to the problem. It explains why the barbaric cruelties of the Tudor period entirely failed to suppress vagrancy, and it points out the only path along which there is a hopeful prospect of ameliorating a great national evil.

### For the Protection of Babies.

It is impossible to exaggerate the importance of diminishing infantile illness and mortality, and every movement having that end in view merits approval. We therefore welcome the announcement that the Lambeth Borough Council are about to open in York Road, Waterloo, a municipal sterilised milk depot, where mothers will not only be able to buy milk for their babies at cost price, but may have them periodically weighed, so that progress can be reported. Arrangements have also been made for the female medical inspectors to visit the homes of those who use the depot milk, in order to see that it is properly used, and that the surroundings of the infant are satisfactory. The need for this action is shown in the fact that the number of infants who annually die in Lambeth alone before they are one year old is 1,400. The scheme, of course, is but another development of similar enterprises elsewhere. In Finsbury, for instance, a depot on the same lines was opened in November, 1904. Dr. Newman, the Medical Officer of Health for that borough, has just published, at an opportune moment, an account of the first year's working. A hundred and twenty-nine babies were entirely supplied with milk, and the results are distinctly encouraging, notwithstanding that, in spite of the elaborate precautions taken, eighteen of the children suffered from infantile diarrhœa, and five of them with fatal consequences. This the medical officer chiefly attributes to the use of what Sir William Broadbent has called "an invention of the devil," in other words, the india-rubber "comforter." Dr. Newman states that these articles, which the children suck and chew at considerable leisure, are dipped in sugary masses or dirty milk to give them a palatable taste, that they fall on the floor and collect dust, that flies settle upon them, that they are only cleansed by a dirty apron, that they are nearly always moist and warm owing to contact with the child's mouth, and that, therefore, they afford an ideal receptacle for the multiplication and development of germ life. No doubt this is true, and the one word we have to say in defence of the comforter is that if only allowed to children at bedtime it helps to send them to sleep, and by keeping the lips closed teaches them to breathe properly through the nose. Of course, it should always be thoroughly cleansed before it is used. Unless that precaution can be strictly observed, it is a perpetual peril to infantile life, which it is the object of milk depots to protect.

